# Racial and ethnic disparities in disease-modifying therapy use among adults with multiple sclerosis: a real-world analysis of the All of Us Research Program

**DOI:** 10.3389/fneur.2026.1916567

**Published:** 2026-09-14

**Authors:** Victoria Fontánez-Ramos, Jonathan Barreto-Nieves, Grecia A. Carrasquillo-Sanabria, Jorge F. García-Báez, Yaritza Inostroza-Nieves

**Affiliations:** Department of Biomedical Sciences, San Juan Bautista School of Medicine, Caguas, PR, United States

**Keywords:** All of Us Research Program, disease-modifying therapy, electronic health records, health disparities, multiple sclerosis, pharmacoepidemiology, race and ethnicity, treatment initiation

## Abstract

**Introduction:**

Racial and ethnic disparities in multiple sclerosis (MS) disease-modifying therapy (DMT) use are documented but incompletely characterized across the full treatment pathway.

**Methods:**

We conducted a retrospective cohort study using the All of Us Research Program (2024 data release) among adults aged ≥18 years with confirmed MS (*N* = 3,508). Outcomes included DMT initiation, time to first DMT, drug class selection, switching, escalation, and discontinuation. High-efficacy DMTs were defined as agents with ≥50% relative reduction in annualized relapse rate. Analyses were descriptive; race and ethnicity were self-reported.

**Results:**

Overall, 1,564 patients (44.6%) initiated DMT. Hispanic patients had the lowest initiation rate (36.2% vs. 45.6% non-Hispanic). Black patients had the highest within-group initiation rate (49.2%) but the longest median time to first DMT (1,497 days vs. 1,217 days in White patients). High-efficacy DMT exposure ranged from 11.9 to 24.0% across race–sex strata, and 76.3% of assessable initiators met the 180-day discontinuation threshold (range 71.7–80.5%).

**Discussion:**

Most patients with MS in this national cohort never initiated DMT. Hispanic patients showed the lowest initiation rates, while Black patients experienced the longest delays to treatment. The two groups’ patterns are consistent with mechanistically distinct barriers, possible limited system entry for Hispanic patients and possible delayed specialty access for Black patients. High-efficacy DMT utilization remained low across all groups. Equitable and timely access to DMT, including high-efficacy agents, is urgently needed to reduce long-term disability across all populations.

## 1 Introduction

Multiple sclerosis (MS) is a chronic autoimmune demyelinating disease of the central nervous system and a leading cause of nontraumatic neurologic disability in young adults (1). An estimated 0.9–1.0 million adults in the United States live with MS, with the disease disproportionately affecting women and individuals aged 20–50 years (2, 3). MS exerts a substantial burden including progressive physical disability, cognitive impairment, and diminished quality of life (4, 5). Disease-modifying therapies (DMTs) target inflammatory mechanisms during the relapsing phase; consequently, early initiation has been associated with reduced relapse activity, delayed disability accumulation, and improved long-term outcomes (6, 7). Recent evidence shows that first-line use of high-efficacy DMTs such as natalizumab, ocrelizumab, alemtuzumab, and cladribine suppresses relapse activity and MRI lesion burden more effectively than platform injectable therapies or the traditional escalation strategy (6–9). Even with the expanding DMT armamentarium, timely and equitable adoption remains incomplete.

Black patients with MS experience greater lesion burden, faster disability accumulation, and worse outcomes than White patients, including faster rates of grey matter, white matter, and thalamic atrophy as well as retinal neurodegeneration on objective neuroimaging (10). Race and ethnicity are not independently associated with disability accumulation after adjustment for individual- and neighborhood-level social determinants of health, suggesting that structural inequities rather than inherent biological differences drive these observed disparities (11). Hispanic patients, one of the fastest-growing MS populations in the United States (2), face barriers including lower insurance coverage, limited specialist access, and language-concordance challenges (12). Prior studies have documented delayed DMT initiation among both groups, and emerging evidence suggests that disparities extend to treatment adherence and discontinuation, with Hispanic and Black patients demonstrating lower persistence on oral and injectable DMTs than non-Hispanic White patients (13). However, whether disparities extend further to drug class selection, escalation to high-efficacy therapy, and switching behavior remains poorly understood (12).

Previous work in a large integrated health system demonstrated that a multicomponent treatment-optimization program could increase high-efficacy DMT use and reduce relapse rates at the health-system level; however, it did not specifically characterize racial and ethnic variation in DMT selection, switching, escalation, or discontinuation across diverse groups (14). A single-center United Kingdom study examined DMT access by ethnicity but was limited in scope and did not assess switching, escalation, or discontinuation patterns (15). Large administrative claims databases such as Optum and MarketScan lack the racial and ethnic granularity needed to adequately represent minority MS populations; race/ethnicity is unknown for approximately 25% of patients, and imputation algorithms have low positive predictive value for Black (40–74%) and Hispanic (52–77%) individuals compared with White individuals (94–95%) (2). The All of Us Research Program, a National Institutes of Health initiative enrolling more than 750,000 participants with purposeful overrepresentation of historically underrepresented groups, electronic health record (EHR) linkage, and a consent-based patient-partnered design, provides a uniquely appropriate platform to address these limitations (16).

The objective of this study was to characterize racial and ethnic differences in real-world MS DMT treatment pathways, including initiation rates, time to first DMT, drug class selection, switching, escalation, and discontinuation, among adults enrolled in the All of Us Research Program. To our knowledge, this is among the first studies to characterize the full real-world DMT treatment pathway across each of these dimensions simultaneously by race and ethnicity in a cohort with purposeful representation of historically underrepresented groups.

## 2 Materials and methods

### 2.1 Study design, cohort, exposures, and outcomes

This was a retrospective cohort study using EHR-derived (real-world) data from the All of Us Research Program, Controlled Tier (workspace aou-rw-2b897985; Controlled Tier Dataset v8; Google Project ID terra-vpc-sc-35650bf1). The study was approved by the Institutional Review Board (IRB) at San Juan Bautista School of Medicine (IRB approval number EMSJBIRB-16-2026). A total of 3,708 individuals with a multiple sclerosis (MS) diagnosis (ICD-10: G35) were initially identified. Eligibility required adults ≥18 years old at first MS diagnosis with at least two MS diagnostic codes (ICD-10: G35) recorded in the EHR and at least 12 months of EHR follow-up after the first MS diagnosis code (17). Exclusion criteria were age <18 at first MS diagnosis code, less than 12 months of EHR follow-up, and other demyelinating diseases without confirmed MS; the MS-only extract pre-screened the latter two criteria, so only the age criterion produced exclusions at the analytic step (*n* = 200), yielding a final analytic cohort of 3,508 adults with confirmed MS.

Two temporal anchors were defined per participant. Because the MS-only extract does not retain pre-extract drug exposures or visit history, the cohort baseline date was set to the earliest recorded clinical activity within the MS-specific extract rather than to the date of MS diagnosis; this baseline served as the analytic time origin for follow-up and time-to-DMT calculations. The MS diagnostic anchor date was the first recorded MS diagnosis code, confirming diagnostic eligibility and post-diagnosis observability. Time-to-DMT estimates therefore reflect time from the earliest MS-extract clinical activity to first observed DMT exposure, not necessarily time from first MS diagnosis.

DMT exposure was evaluated relative to the cohort baseline date. The analytic extract contained a pre-baseline DMT indicator; however, this flag was structurally zero for all participants in the MS-only extract. The DMT initiation outcome therefore represents the first observed post-baseline DMT exposure within the available EHR data, not necessarily the first lifetime DMT exposure. Participants without a post-baseline DMT record were classified as non-initiators during follow-up. Because the MS-only extract does not retain pre-extract drug exposure history, we cannot distinguish DMT-naïve patients from prevalent users whose treatment began before cohort entry and continued across the baseline date; this ascertainment limitation, common to left-truncated EHR cohorts, should be considered when interpreting initiation rates and time-to-treatment estimates. DMT exposures were ascertained using a concept-based mapping framework that identified 132 drug concepts from the Observational Medical Outcomes Partnership Common Data Model (OMOP CDM) drug_exposure table, encompassing approved and commonly used MS disease-modifying agents available during the study period; the full list of OMOP concept identifiers is provided in Supplementary material. Identified drugs were classified into five therapeutic classes: (1) injectable agents (glatiramer acetate, interferon-beta products); (2) oral moderate-efficacy agents (dimethyl fumarate, teriflunomide); (3) oral high-efficacy agents (siponimod, cladribine); (4) infused high-efficacy agents (natalizumab, ocrelizumab, alemtuzumab); and (5) off-label historical agents (mitoxantrone and other cytotoxic agents). High-efficacy DMTs were defined *a priori* as agents demonstrating ≥50% relative reduction in annualized relapse rate in pivotal clinical trials (6, 7, 9). This threshold follows the classification used in prior large-scale observational studies of DMT timing and efficacy (6), in which agents demonstrating approximately ≥50% relative ARR reduction versus placebo or active comparator in pivotal trials, natalizumab (AFFIRM), ocrelizumab (OPERA I/II), alemtuzumab (CARE-MS I/II), and cladribine (CLARITY), are classified as high-efficacy, in contrast to platform injectable and first-generation oral agents demonstrating lower relative ARR reductions. Rituximab was classified among the infused high-efficacy agents in the primary analysis, given its B-cell–depleting mechanism shared with ocrelizumab and its widespread real-world use throughout the study period, despite its off-label status for MS.

DMT exposure end dates were assigned hierarchically: directly from the drug_exposure_end_datetime field (79.5% of records), imputed from the days_supply field when the direct date was absent (14.4%), or classified as unassessable when neither source was available (6.1%). End-date source distributions stratified by DMT class are summarized in Supplementary Table S4. The primary exposure groups for comparative analyses were defined by race (White, Black, Other) and ethnicity (Hispanic, Non-Hispanic), treated as separate but related dimensions given their substantial overlap. The “Other” race category was a residual grouping comprising all participants not classified as White or Black. Secondary stratification was performed by sex and by three calendar eras: 2000–2009, 2010–2016, and 2017 onward. Because participants entering later calendar eras had less calendar time available for follow-up, lower initiation proportions in the 2017+ era may partly reflect administrative censoring rather than true reductions in treatment uptake.

The primary outcomes were: (1) DMT initiation status; (2) time from cohort baseline to first DMT, summarized as mean and median given the right-skewed distribution; (3) first DMT class; (4) switching, defined as a documented change from one DMT to a different DMT within the same patient’s observed exposure sequence; (5) escalation, defined as a switch from a moderate-efficacy class (injectable or oral moderate-efficacy) to a high-efficacy class (oral high-efficacy or infused high-efficacy); (6) de-escalation, defined as the reverse direction; and (7) discontinuation, defined as a gap exceeding 180 days beyond the expected exposure end date. The expected exposure end date was the recorded drug_exposure_end_datetime when present, or the start date plus days_supply when absent; records with neither were excluded from the discontinuation analysis. Time to first DMT was additionally categorized into four bins: ≤90 days, 91–365 days, >365 days, and never initiated.

All-cause healthcare utilization during the 365 days following cohort baseline was summarized across outpatient, inpatient, and emergency department categories. Comorbidity burden was assessed using a rule-based keyword classification on condition concept names, organized into hypertension, cardiovascular disease, diabetes, and a composite “other” category. Cells with fewer than 20 participants were suppressed per All of Us Controlled Tier reporting requirements. As a consequence of this *n* < 20 cell-suppression rule, sex-stratified counts in some figures and tables do not sum to the cohort total of 3,508; the unreported residual comprises Unknown/Other sex patients (*n* = 13 cohort-wide; Supplementary Table S7) plus any Female or Male sub-stratum whose displayed cell falls below the suppression threshold.

### 2.2 Statistical analysis

All analyses were descriptive; no multivariable regression models, confidence intervals, or inferential *p*-values were reported. The primary aim was to characterize the MS treatment landscape across racial, ethnic, sex, and calendar-era strata rather than to estimate causal effects. Because analyses were descriptive and unadjusted, observed differences across race, ethnicity, and sex strata cannot be interpreted as independent effects of these variables; they may reflect the influence of unmeasured confounders including insurance status, socioeconomic status, specialist access, disease severity, and MRI lesion burden. Adjusted analyses examining independent predictors of DMT initiation and time-to-treatment are planned for future work. The association between race and ethnicity was quantified using a chi-square test solely to derive Cramér’s *V* as an effect-size measure (Cramér’s *V* = 0.703; 89.9% of Hispanic participants belonged to the “Other” race group); the resulting *p*-value was not used for inferential decision-making and is not reported. Chi-square tests were not applied to between-group outcome comparisons. Given the right-skewed distribution of time to first DMT, the median was the primary summary measure.

Three pre-specified sensitivity analyses were conducted. First, discontinuation was re-evaluated using 90-day and 365-day gap thresholds (Supplementary Table S5). Second, rituximab was reclassified as a separate off-label agent category to assess the sensitivity of efficacy-class estimates to this classification decision; results were qualitatively unchanged. Third, primary outcomes were re-examined restricted to participants with a cohort baseline date on or after 1 January 2017, to evaluate whether pooling across treatment eras influenced the reported race and ethnicity differences (Supplementary Table S8). All prespecified data-integrity checks and analytic quality gates passed without violation, including cohort timeline consistency (*n* = 3,508), DMT sequence construction (*n* = 44,983 exposures), and baseline-definition audits across all analysis sections.

## 3 Results

### 3.1 Cohort characteristics

The final analytic cohort comprised 3,508 adults with confirmed MS. The cohort was predominantly female (*n* = 2,714; 77.4%), consistent with the established 3:1 female-to-male epidemiology of MS, with 781 male participants (22.3%) and 13 (0.4%) with sex recorded as Unknown/Other. By self-reported race, 2,396 (68.3%) were White, 557 (15.9%) were Black, and 555 (15.8%) were classified as Other; by ethnicity, 376 (10.7%) identified as Hispanic. Race and ethnicity categories showed strong collinearity: 89.9% of Hispanic patients fell within the Other-race group (Cramér’s *V* = 0.703; Supplementary Table S2). Baseline cohort characteristics, including comorbidity burden and healthcare utilization, are presented in Table 1.

**Table 1:** Baseline characteristics of the study cohort, overall and by race and ethnicity.

Characteristic	Overall (*N* = 3,508)	White (*n* = 2,396)	Black (*n* = 557)	Other (*n* = 555)	Hispanic (*n* = 376)	Non-Hispanic (*n* = 3,132)
Female, *n* (%)	2,714 (77.4)	—	—	—	—	—
Male, *n* (%)	781 (22.3)	—	—	—	—	—
Hispanic ethnicity, *n* (%)	376 (10.7)	31 (1.3)	— (suppressed, *n* < 20)	338 (60.9)	—	—
Mean comorbidity burden score	2.05	1.97	2.48	1.97	1.97	2.06
Hypertension, %	48.2	44.9	65.0	45.6	45.7	48.5
Cardiovascular disease, %	34.8	34.3	41.8	30.1	28.2	35.6
Diabetes, %	21.3	18.1	31.4	24.7	27.4	20.5
≥1 non-MS comorbidity, %	67.3	—	—	—	—	—
Migraine, %	22.5	—	—	—	—	—
Mean total visits (365 d)	11.1	10.9	11.5	11.8	10.7	11.2
Mean outpatient visits	10.2	10.1	10.3	10.6	9.5	10.3
Mean inpatient visits	0.51	0.49	0.46	0.66	0.63	0.50
Mean ED visits	0.39	0.30	0.76	0.60	0.56	0.40

Data are *n* (%) or mean unless otherwise indicated. Sex and selected cohort-level values are presented for the overall cohort; race- and ethnicity-specific cells reflect within-group values. Dash (—) indicates value not stratified by this variable in the source analysis or cell suppressed in accordance with the All of Us Data and Statistics Dissemination Policy (*n* < 20). The comorbidity burden score reflects the count of rule-based condition groups identified from condition concept names. CVD, cardiovascular disease; ED, emergency department; MS, multiple sclerosis.

### 3.2 DMT initiation

Of 3,508 patients, 1,564 (44.6%) initiated any DMT during the observation period. Because DMT exposure was ascertained from EHR-linked drug_exposure records, this estimate should be interpreted as a lower bound on true treatment uptake if patients filled prescriptions through channels not captured in the linked EHR data; conversely, the discontinuation estimate reported below may overstate true treatment cessation if patients continued therapy outside the observable network. Black patients had the highest within-group initiation rate (49.2%), followed by White (45.3%) and Other-race patients (36.8%). Hispanic patients had the lowest initiation rate of any group (36.2%) compared with non-Hispanic patients (45.6%). Black patients also exhibited the longest median time to first DMT (1,497 days, vs. 1,217 days in White and 1,329 days in Other-race patients). Among initiators, Hispanic patients had a median time to first DMT (1,206 days) comparable to, and modestly shorter than, non-Hispanic patients (1,320 days). Rapid starts within 90 days were uncommon across all groups, and non-initiation remained the most frequent outcome in every race–sex and ethnicity stratum, reaching its highest level in the Other-race group (63.2%; Figure 1; Supplementary Table S3).

**Figure 1:**
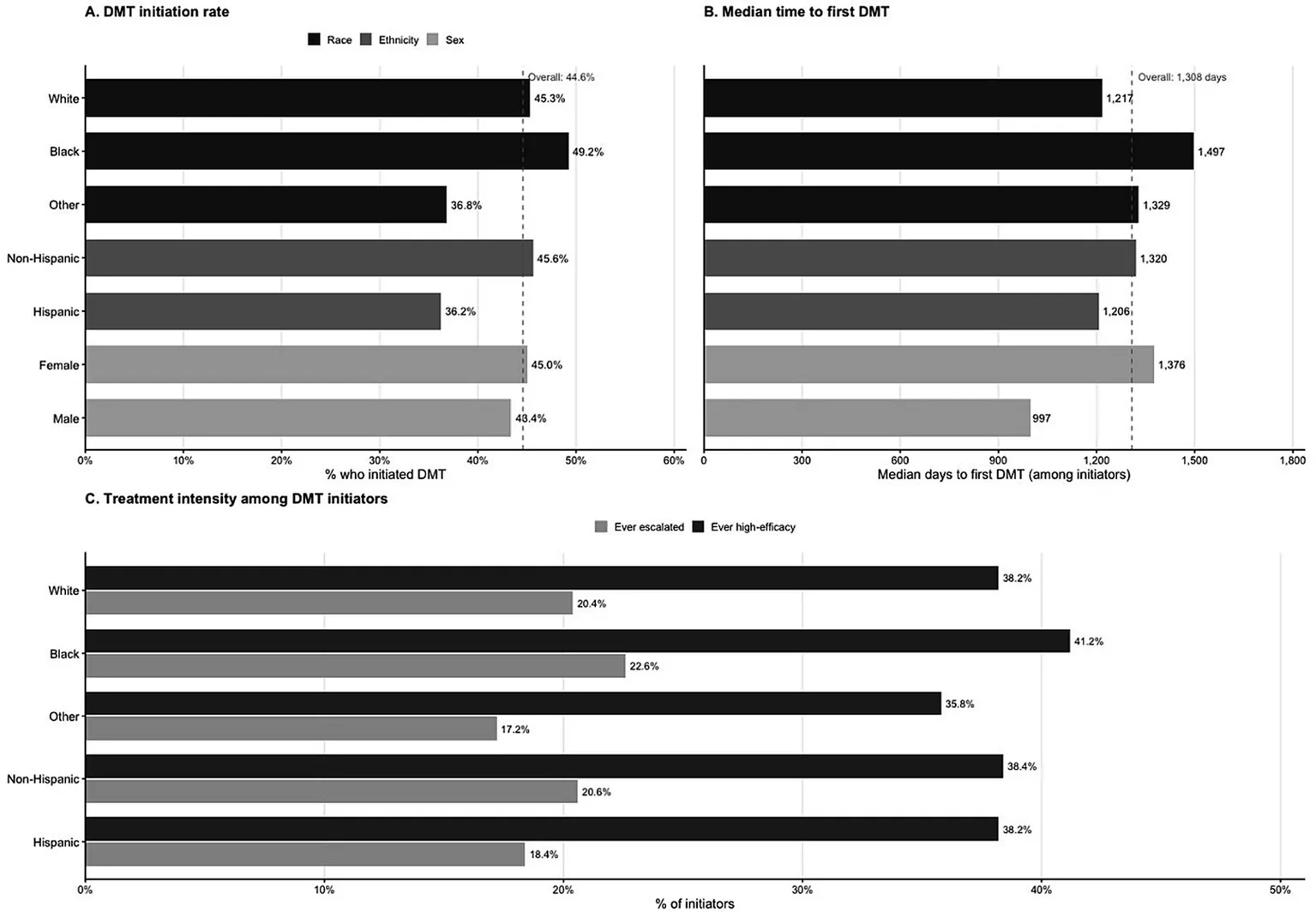
Disease-modifying therapy (DMT) initiation, time to first DMT, and treatment intensity by race, ethnicity, and sex among adults with multiple sclerosis in the All of Us Research Program (*N* = 3,508). Panel A shows the percentage of patients within each group who initiated any DMT during follow-up. Panel B shows the median number of days from cohort baseline to first observed DMT exposure among initiators. Panel C shows the percentage of initiators who ever received any high-efficacy DMT and who ever escalated to a higher-efficacy class, stratified by race and ethnicity; sex-stratified estimates were not available for these endpoints. Bars are shaded in grayscale by grouping dimension (Race, Ethnicity, Sex). Cohort total *N* = 3,508 (Female *n* = 2,714; Male *n* = 781; Unknown/Other *n* = 13). Unknown/Other-sex cells are suppressed in stratified analyses in accordance with the All of Us Data and Statistics Dissemination Policy, which requires suppression of any displayed cell with n < 20. DMT, disease-modifying therapy.

### 3.3 First DMT class and temporal trends

Among initiators, injectable agents were the most common first-line class overall (51.2%; Table 2), while oral high-efficacy agents were rarely used as first-line therapy (cells suppressed for *n* < 20 in all race strata). Infused high-efficacy DMTs were associated with the shortest median time to initiation, consistent with preferential selection of these agents in patients who entered specialty care earlier or presented with more aggressive disease.

**Table 2:** First DMT class distribution among DMT initiators, by race.

First DMT class	White (*n* = 1,086)	Black (*n* = 274)	Other (*n* = 204)
Injectable, *n* (%)	556 (51.2)	148 (54.0)	97 (47.5)
Oral moderate-efficacy, *n* (%)	269 (24.8)	53 (19.3)	50 (24.5)
Infused high-efficacy, *n* (%)	206 (19.0)	50 (18.2)	38 (18.6)
Off-label historical, *n* (%)	48 (4.4)	21 (7.7)	— (suppressed, *n* < 20)
Oral high-efficacy, *n* (%)	— (suppressed, *n* < 20)	— (suppressed, *n* < 20)	— (suppressed, *n* < 20)

Denominators are DMT initiators within each race group. Cells with *n* < 20 were suppressed in accordance with the All of Us Data and Statistics Dissemination Policy. DMT, disease-modifying therapy.

A clear temporal shift was observed across calendar eras (Figure 2): median time to first DMT decreased from 3,092 days in 2000–2009 to 184 days in 2017+, and the most common first-line class shifted from injectable agents in earlier eras to infused high-efficacy DMTs in 2017+. The apparent decline in within-era initiation rate in 2017+ (31.7%) likely reflects truncated observation time rather than a true reduction in treatment uptake, as participants in the most recent era have had less follow-up time to initiate therapy.

**Figure 2:**
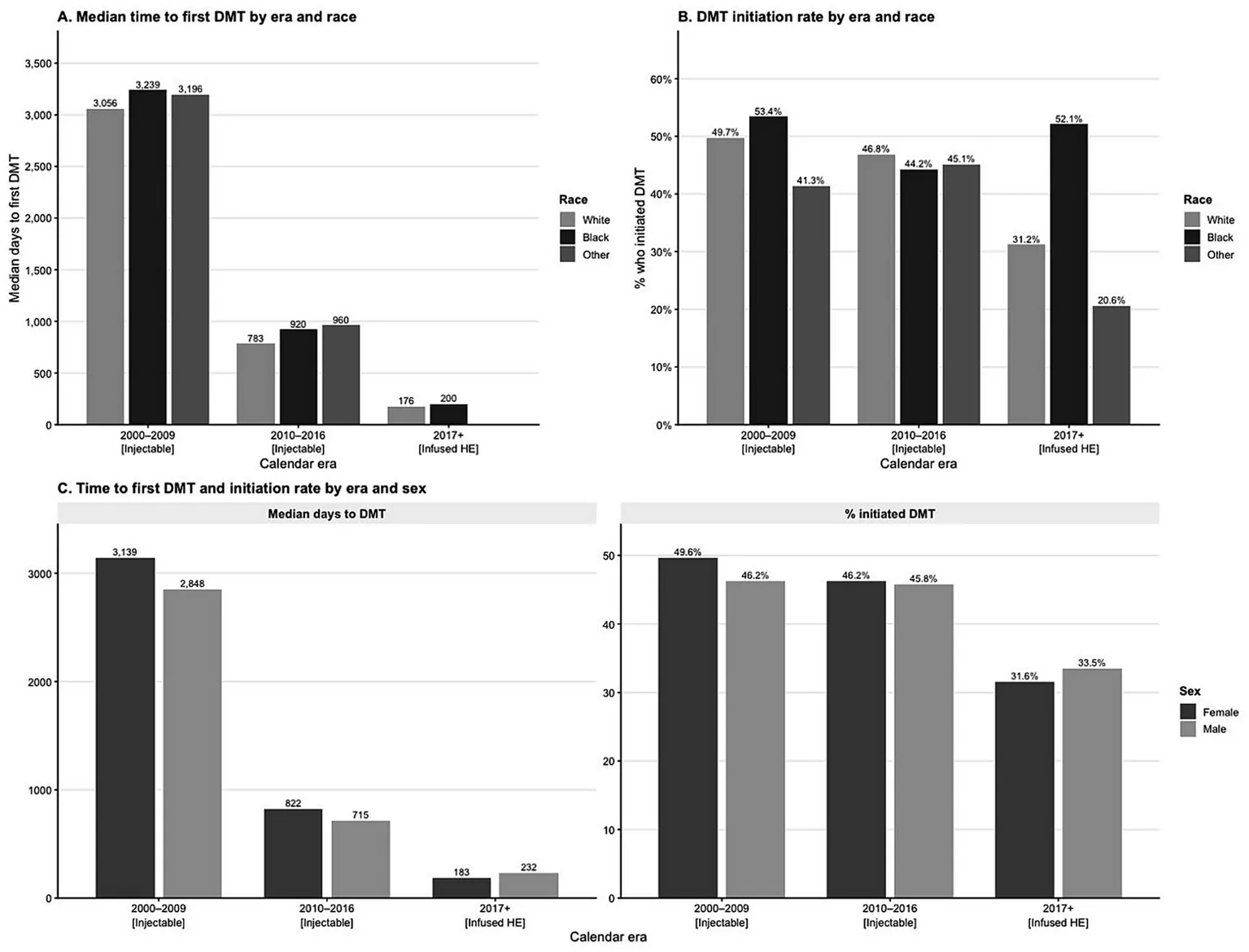
DMT initiation patterns across three calendar eras (2000–2009; 2010–2016; 2017+) by race and sex among adults with multiple sclerosis in the All of Us Research Program (*N* = 3,508). **(A)** Shows median days to first DMT by era and race. **(B)** Shows the percentage of patients initiating DMT by era and race. **(C)** Shows median days to first DMT (left facet) and initiation rate (right facet) by era and sex. Bracketed labels below each era indicate the most common first-line DMT class observed in that era. The Other-race 2017+ median (*n* = 32 initiators) is suppressed because the assessable subset for median calculation fell below the suppression threshold under the All of Us data-dissemination policy. DMT, disease-modifying therapy.

To assess whether pooling heterogeneous treatment eras influenced the observed racial differences, we repeated the initiation analysis restricted to participants with a cohort baseline date on or after 1 January 2017 (*n* = 650; Supplementary Table S8). Restriction to the contemporary era did not attenuate between-group differences in initiation. The Black–White difference in initiation rate increased from 3.9 percentage points in the full cohort to 21.0 percentage points in the 2017+ subcohort, and the Black–Other-race difference increased from 12.4 to 31.5 percentage points, with initiation reaching 52.1% among Black, 31.2% among White, and 20.6% among Other-race participants. Infused high-efficacy agents were the most common first-line class in all three race groups in this period. Because absolute initiation rates within the 2017+ subcohort are depressed by truncated observation time, the interpretable quantity is the between-group difference within the era rather than its absolute level.

### 3.4 High-efficacy DMT exposure

Overall high-efficacy DMT exposure, including both first-line high-efficacy initiation and escalations from lower-efficacy agents, remained low across all race–sex strata despite the growing evidence base supporting early intensive treatment. Median time to high-efficacy DMT exceeded 4.8 years in all assessable strata, with the longest delays observed in Black female patients (median 7.1 years), followed by Other-race female patients (6.6 years). Black male patients showed the shortest median time to high-efficacy DMT among the assessable strata (4.8 years; Supplementary Table S1).

### 3.5 Switching, escalation, and discontinuation

Switching was somewhat more common in Black patients (42.3%) than in White (39.6%) or Other-race (31.4%) patients; Hispanic patients switched less frequently than non-Hispanic patients (31.6% vs. 39.7%). Escalation to high-efficacy therapy occurred in 17.2–22.6% of initiators across groups, indicating that the majority who started on lower-efficacy therapy never progressed to higher-efficacy agents. De-escalation was uncommon across all groups (<10%). Overall, 76.3% of assessable initiators (957/1,254) met the 180-day discontinuation definition (Supplementary Table S5); by race/ethnicity, this ranged from 71.7 to 80.5% (Table 3). Reclassifying rituximab as a separate off-label agent in sensitivity analysis did not qualitatively alter these findings.

**Table 3:** Treatment patterns, escalation, and discontinuation among DMT initiators, by race and ethnicity.

Outcome	White (*n* = 1,086)	Black (*n* = 274)	Other (*n* = 204)	Hispanic (*n* = 136)	Non-Hispanic (*n* = 1,428)
Ever switched DMT, %	39.6	42.3	31.4	31.6	39.7
Ever escalated to high efficacy, %	20.4	22.6	17.2	18.4	20.6
Ever de-escalated, %	9.1	5.1	3.9	2.2	8.3
Ever received high-efficacy DMT, %^a^	38.2	41.2	35.8	38.2	38.4
Assessable for discontinuation, *n*	865	219	170	118	1,136
Discontinued (180-day gap), %^b^	76.9	71.7	79.4	80.5	75.9
Median time to discontinuation, days	2,082	2,104	1,550	1,282	2,111

Denominators are DMT initiators within each group. Race and ethnicity columns are not mutually exclusive; 89.9% of Hispanic participants are classified within the Other-race group (Cramér’s *V* = 0.703). ^a^Includes high-efficacy exposure via first-line initiation or escalation. ^b^Percentage of the assessable subset only (see Methods 2.1); non-assessable initiators excluded from denominator.

### 3.6 Healthcare utilization and comorbidity burden

Emergency department utilization was more than twice as high in Black patients as in White patients (0.76 vs. 0.30 mean visits per year), a pattern consistent with episodic rather than longitudinal disease management. Black patients also carried the highest rule-based comorbidity burden score (mean 2.48 vs. 1.97 in both White and Other-race groups), with substantially higher prevalence of hypertension, cardiovascular disease, and diabetes than other groups (Table 1).

## 4 Discussion

In this real-world analysis of 3,508 adults with MS from the All of Us Research Program, fewer than half of patients initiated DMT, with differences observed across the treatment pathway by race and ethnicity in initiation, timing, class selection, and discontinuation. The cohort was predominantly female, consistent with the known epidemiology of MS (1, 2), and its demographic diversity reflects the Program’s intentional oversampling of historically underrepresented populations, which enabled more robust evaluation of disparities than is typically possible in conventional cohorts (16). Hispanic patients had the lowest overall DMT initiation rate, whereas Black patients demonstrated the highest within-group initiation but faced the longest delays to treatment, and high-efficacy DMT utilization remained limited across all subgroups (Figure 1). Because insurance status, socioeconomic status, specialist access, disease severity, and MRI lesion burden were not captured in this dataset, all interpretations regarding underlying mechanisms offered below are hypothesis-generating and should not be interpreted as evidence of independent or causal effects of race or ethnicity.

### 4.1 Comparison with prior literature and racial differences in treatment access

Our findings extend previously documented racial and ethnic disparities in MS care. Prior studies have established that Black and Hispanic patients with MS face substantial inequities, but most focused on isolated metrics within single health systems (12, 14, 18). To our knowledge, this is among the first studies to use the All of Us Research Program to characterize the complete MS treatment pathway across racial and ethnic groups. A recent United Kingdom study similarly found that non-White patients were less likely to initiate DMT or a high-efficacy agent (15), and the gaps we observed, a 9.4-percentage-point initiation difference between Hispanic and non-Hispanic patients and a median delay of approximately 280 days between Black and White patients (Figure 1, confirm that these patterns extend well beyond a single health system.

These patterns are consistent with, though do not confirm, two different barriers. For Black patients, the gap appears to lie in how quickly treatment begins rather than whether it begins at all: their within-group initiation rate exceeded that of White patients, but delays were substantially longer. The combination of the highest within-group initiation rate, the longest median time to first DMT (Figure 1), and the highest comorbidity burden (Table 1) could be consistent with delayed referral into MS specialty care, although disease severity, specialist density, and insurance status were not measured and cannot be ruled out as contributors. The Hispanic pattern is different. Median time to first DMT among Hispanic initiators was comparable to, and modestly shorter than, that of non-Hispanic patients (Figure 1), so the disparity sits at the point of initial entry into care rather than at the prescribing step. Closing that gap will require upstream work on identification and referral rather than faster prescribing once patients arrive (19). Elevated emergency department utilization in Black patients, with modestly elevated utilization in Hispanic patients (Table 1), alongside lower or delayed DMT access, is in keeping with a pattern of fragmented, episodic care (12).

Hispanic patients carried a dual burden that no other group in the cohort showed: the lowest initiation rate together with the highest observed discontinuation rate. Their mean comorbidity burden score was identical to that of White and Other-race patients (Table 1), yet initiation remained the lowest. Because Hispanic patients’ mean comorbidity burden score was similar to White and Other-race patients rather than resembling the higher-burden Black patient group, clinical complexity alone does not appear to explain the lower initiation rate in this subgroup. A structural explanation is one plausible interpretation of these data, though it cannot be distinguished from unmeasured factors such as insurance status or specialist access, and barriers may operate at diagnosis, referral, and system entry rather than at the prescribing step. This interpretation is hypothesis-generating, because insurance, language preference, and geographic distance to MS specialty care were not measured.

One finding inside the Black patient group runs against the overall picture of undertreatment. Black male patients had the highest within-group rate of high-efficacy DMT initiation in the cohort (24.0%). A plausible reading is clinical rather than equitable: Black patients with MS tend to present with higher inflammatory disease activity, and recent guidelines increasingly favor high-efficacy first-line therapy in active relapsing disease (1, 3). If Black men in this cohort disproportionately presented with more aggressive disease, the elevated high-efficacy rate may track disease severity rather than parity of access.

### 4.2 Low high-efficacy DMT utilization and the cost of delay

Injectable agents dominated as first-line therapy (Figure 2) and cumulative high-efficacy exposure remained low across every race and sex stratum, despite a strong evidence base for early intensive treatment in relapsing MS (3, 6, 20). Early high-efficacy therapy has been associated with lower 5-year disability progression and an approximately 55% lower hazard of relapse compared with moderate-efficacy initiation (6, 21). Because axonal loss and neurodegeneration begin before disability becomes clinically apparent (1), delayed or insufficient treatment can translate into irreversible neurological injury. The combination of low overall initiation, long delays among Black patients, and limited high-efficacy uptake across groups indicates under-utilization of high-efficacy DMTs relative to current guideline recommendations (20, 22), with direct consequences for early disease modification within the relapsing phase.

Median time to first DMT fell from 3,092 days in 2000–2009 to 184 days in 2017 onward, and infused high-efficacy agents became the most common first-line class in the most recent era (Figure 2). This shift coincides temporally with the March 2017 FDA approval of ocrelizumab, the first DMT approved for primary progressive MS; temporal coincidence alone cannot establish a causal contribution, but the timing is consistent with a role for this approval in the observed shift toward infused high-efficacy first-line therapy. The era-level improvement, however, did not erase between-group disparities and should not be read as resolution of the underlying access gap.

### 4.3 Strengths and limitations

Strengths of this study include the large, nationally diverse cohort with representation of historically underrepresented populations (16, 23) and the ability to evaluate multiple stages of the treatment pathway, providing a more comprehensive picture of disparities than prior single-outcome studies.

Several limitations warrant consideration. The index date was based on the earliest recorded MS-related activity rather than confirmed diagnosis, which may inflate apparent time-to-DMT. Because pre-baseline treatment history could not be ascertained, initiation and time-to-DMT estimates likely reflect a mixture of incident and prevalent DMT use rather than incident use alone. Race and ethnicity showed strong collinearity between the Other-race and Hispanic groups (89.9% overlap; Cramér’s *V* = 0.703). Comorbidities were identified by rule-based matching rather than validated phenotyping, and end-date information was unavailable for 6.1% of DMT exposures and imputed for 14.4%. MS phenotype could not be characterized in this cohort. The cohort was defined using ICD-10 code G35, which is not subdivided by clinical course, and the All of Us Controlled Tier does not include free-text clinical documentation from which phenotype might otherwise be inferred. We therefore cannot report the proportion of participants with progressive disease, nor determine whether phenotype influenced treatment eligibility. This is a material limitation, because primary and secondary progressive MS carry substantially narrower approved treatment options than relapsing forms, and any between-group difference in phenotype distribution would contribute to the observed differences in initiation and class selection independently of access. JCV serostatus, MRI data, and clinical severity measures were likewise unavailable, so whether prescribing differences reflect clinical appropriateness or access inequity cannot be fully disentangled. Unmeasured structural factors, including insurance status, language preference, geography, MS specialist density, pregnancy planning, and adherence, likely explain a meaningful share of between-group variation but could not be adjusted for. The study period spans more than two decades of substantial change in MS therapeutics, and pooling calendar eras could in principle distort between-group comparisons; a sensitivity analysis restricted to participants entering the cohort in 2017 or later showed that racial differences in initiation widened rather than narrowed (Supplementary Table S8), indicating that era pooling is unlikely to account for the reported disparities, though this restricted analysis could not be extended to ethnicity strata, escalation, or discontinuation because of cell-suppression thresholds and insufficient follow-up within the 2017+ window. Finally, these findings are derived from a US-based population enrolled in a US research program with a distinct healthcare financing and specialty-access structure; while the qualitative pattern of racial and ethnic disparity is consistent with findings from a UK-based study (15), the specific magnitudes reported here may not generalize to healthcare systems with different insurance, referral, or DMT-access structures.

Two pharmacoepidemiologic caveats apply. First, this is a descriptive analysis without adjustment for confounding by indication; patients receiving high-efficacy first-line therapy differ systematically from those on platform injectables in disease severity, diagnosis recency, and specialist access. Second, the discontinuation variable captures an EHR treatment gap rather than a confirmed stop, and may include payer disruption, switches outside the All of Us network, or pregnancy-related pauses. For the same reason, the low overall initiation rate (44.6%) should be interpreted as a lower bound on true treatment uptake rather than a definitive estimate. All analyses are descriptive; causal relationships cannot be inferred.

## 5 Conclusion

Racial and ethnic disparities in MS treatment pathways in this cohort were substantial. The pattern observed is consistent with two distinct types of barriers: for Black patients, structural delays that slowed entry into treatment despite ultimately higher initiation rates; for Hispanic patients, barriers associated with limited treatment entry overall and the highest observed discontinuation rates. If confirmed by studies incorporating insurance, specialist-access, and severity data, these distinct patterns would call for different interventions. High-efficacy DMT use remained limited across all groups at exactly the stage of disease when early intervention matters most, while axonal damage accrues before disability becomes clinically visible. Closing these gaps will require targeted upstream strategies for Hispanic patients, focused on diagnosis, referral, and system entry, together with measures to accelerate specialist access for Black patients, so that the efficacy gains documented in clinical trials translate into equitable real-world care.

## Data Availability

The original contributions presented in the study are included in the article/Supplementary material, further inquiries can be directed to the corresponding authors.
