# Supplementary material for: Racial and ethnic disparities in disease-modifying therapy use among adults with multiple sclerosis: a real-world analysis of the All of Us Research Program
Source: Front Neurol. 2026 Sep 14;17:1916567. doi: 10.3389/fneur.2026.1916567 (PMC13616653; doi:10.3389/fneur.2026.1916567)
Supplement: Supplementary file 1 [file Table_1.DOCX]

Supplementary Tables

**Supplementary Table S1.** Overall high-efficacy DMT exposure by race × sex among Black and Other-race participants.

| **Race** | **Sex** | **N** | **High-efficacy initiated N** | **Median days to high-efficacy DMT** | **% who initiated high efficacy** |
| --- | --- | --- | --- | --- | --- |
| Black | Female | 457 | 89 | 2,594 | 19.5 |
| Black | Male | 100 | 24 | 1,749 | 24.0 |
| Other | Female | 430 | 51 | 2,416 | 11.9 |
| Other | Male | 117 | 21 | 2,114 | 17.9 |

*Among everyone in the Black and Other-race strata whether they initiated DMT, this table reports how many ever received a high-efficacy DMT at any point. The denominator includes both patients who started high efficacy as their first drug and those who escalated to it later, broken down by race and sex. Overall high-efficacy DMT exposure was low across all strata, ranging from 11.9% (Other/Female) to 24.0% (Black/Male). Black patients showed the highest high-efficacy exposure rates in both sexes, a pattern that may reflect more aggressive disease presentation prompting escalation, or eventual access to high-efficacy therapy that occurred with longer delays; median time to high-efficacy DMT was substantial across all strata (1,749–2,594 days for Black patients and 2,114–2,416 days for Other-race patients). Race-level high-efficacy DMT exposure for White participants is reported in Table 2B (ever received high-efficacy DMT: 38.2%) and is not separately tabulated by sex in this supplementary table. These exposure estimates include both first-line high-efficacy starts and escalations from other agents and should therefore be interpreted as "overall high-efficacy DMT exposure" rather than a first-line high-efficacy strategy. DMT, disease-modifying therapy.*

**Supplementary Table S2.** Cross-tabulation of race and ethnicity (n=3,508).

(A) Cross tabulation of race and ethnicity

| **Race group** | **Hispanic** | **Non-Hispanic** | **Total** |
| --- | --- | --- | --- |
| Black | — (suppressed, n<20) | 550 | 557 |
| Other | 338 | 217 | 555 |
| White | 31 | 2,365 | 2,396 |
| Total | 376 | 3,132 | 3,508 |

(B) Association statistics

| **Statistic** | **Value** | **Interpretation** |
| --- | --- | --- |
| Cramér’s V | 0.703 | Very strong association |
| Degrees of freedom | 2 | 3 race groups × 2 ethnicity groups |
| N | 3,508 | Total cohort |

*The chi-square test was used solely to derive Cramér’s V as an effect-size measure; the resulting p-value was not used for inferential decision-making and is not reported. Cells with n<20 were suppressed in accordance with the All of Us Data and Statistics Dissemination Policy.*

**Supplementary Table S3.** Time-to-first-DMT delay categories, by race × sex and by ethnicity.

(A) Delay categories by race and sex.

| **Race** | **Sex** | **≤90 days, n (%)** | **91–365 days, n (%)** | **>365 days, n (%)** | **Never, n (%)** | **Total, n** |
| --- | --- | --- | --- | --- | --- | --- |
| Black | Female | 26 (5.7) | 20 (4.4) | 184 (40.3) | 227 (49.7) | 457 |
| Black | Male | — | — | 37 (37.0) | 55 (55.0) | 100 |
| Other | Female | 28 (6.5) | — | 104 (24.2) | 281 (65.3) | 430 |
| Other | Male | — | — | 39 (33.3) | 59 (50.4) | 117 |
| White | Female | 121 (5.8) | 108 (5.2) | 625 (29.9) | 1,239 (59.2) | 2,093 |
| White | Male | 63 (21.7) | 34 (11.7) | 117 (40.3) | 76 (26.2) | 290 |

(B) Delay categories by ethnicity.

| **Ethnicity** | **≤90 days, n (%)** | **91–365 days, n (%)** | **>365 days, n (%)** | **Never, n (%)** |
| --- | --- | --- | --- | --- |
| Hispanic (n=376) | 28 (7.4) | — | 93 (24.7) | 240 (63.8) |
| Non-Hispanic (n=3,132) | 185 (5.9) | 147 (4.7) | 1,096 (35.0) | 1,704 (54.4) |

*Percentages are computed within each row (race-sex or ethnicity group). Rows for participants with sex recorded as Unknown or Other (n=21) are omitted because all sub-cells fell below the suppression threshold; these participants are included in by-race totals. Cells with n<20 were suppressed per the All of Us Data and Statistics Dissemination Policy.*

**Supplementary Table S4.** DMT exposure end-date source distribution, overall and by DMT class.

| **DMT class** | **Total exposures, n** | **Direct end date, n (%)** | **Days-supply imputation, n (%)** | **Unassessable, n (%)** |
| --- | --- | --- | --- | --- |
| Injectable | 18,457 | 14,234 (77.1) | 3,527 (19.1) | 696 (3.8) |
| Infused high efficacy | 14,080 | 12,046 (85.6) | 796 (5.7) | 1,238 (8.8) |
| Oral / off-label classes (combined)a | 12,446 | 9,494 (76.3) | 2,133 (17.1) | 819 (6.6) |
| **All classes** | **44,983** | **35,774 (79.5)** | **6,456 (14.4)** | **2,753 (6.1)** |

*End dates were assigned hierarchically: directly from the drug_exposure_end_datetime field when present, imputed from the days_supply field when the direct date was absent, or classified as unassessable when neither source was available. a Combined row aggregates oral moderate-efficacy, oral high-efficacy, and off-label historical agents because per-class breakdowns were not separately tabulated for these classes. DMT, disease-modifying therapy.*

**Supplementary Table S5.** Discontinuation sensitivity analysis across gap thresholds.

| **Gap threshold** | **N assessable** | **N discontinued** | **Discontinued, %** |
| --- | --- | --- | --- |
| 90 days | 1,254 | 1,071 | 85.4 |
| **180 days (primary analysis)** | **1,254** | **957** | **76.3** |
| 365 days | 1,254 | 836 | 66.7 |

*Discontinuation was defined a priori as a gap exceeding a specified threshold beyond the expected exposure end date. Participants assessable for discontinuation required either a recorded drug-exposure end datetime or a calculable end date from days_supply. The 180-day threshold corresponds to the primary analysis reported in the main text. Median time to discontinuation was relatively stable across thresholds (range, approximately 1,463–1,529 days). DMT, disease-modifying therapy.*

**Supplementary Table S6.** DMT initiation patterns by calendar era, sex, and race.

(A) Overall, by calendar era.

| **Calendar era** | **Cohort N** | **Initiated, n (%)** | **Median days to DMT** | **Most common first class** |
| --- | --- | --- | --- | --- |
| 2000–2009 | 1,397 | 684 (49.0) | 3,092 | Injectable |
| 2010–2016 | 1,461 | 674 (46.1) | 817 | Injectable |
| 2017+ | 650 | 206 (31.7) | 184 | Infused high-efficacy |

(B) By sex and calendar era

| **Calendar era** | **Sex** | **Cohort N** | **Initiated, n (%)** | **Median days to DMT** |
| --- | --- | --- | --- | --- |
| 2000–2009 | Female | 1,090 | 541 (49.6) | 3,139 |
| 2000–2009 | Male | 301 | 139 (46.2) | 2,848 |
| 2010–2016 | Female | 1,144 | 528 (46.2) | 822 |
| 2010–2016 | Male | 310 | 142 (45.8) | 715 |
| 2017+ | Female | 471 | 149 (31.6) | 183 |
| 2017+ | Male | 170 | 57 (33.5) | 232 |

(C) By race and calendar era.

| **Calendar era** | **Race** | **Cohort N** | **Initiated, n (%)** | **Median days to DMT** |
| --- | --- | --- | --- | --- |
| 2000–2009 | White | 956 | 475 (49.7) | 3,056 |
| 2000–2009 | Black | 223 | 119 (53.4) | 3,239 |
| 2000–2009 | Other | 218 | 90 (41.3) | 3,196 |
| 2010–2016 | White | 1,039 | 486 (46.8) | 783 |
| 2010–2016 | Black | 240 | 106 (44.2) | 920 |
| 2010–2016 | Other | 182 | 82 (45.1) | 960 |
| 2017+ | White | 401 | 125 (31.2) | 176 |
| 2017+ | Black | 94 | 49 (52.1) | 200 |
| 2017+ | Other | 155 | 32 (20.6) | — |

*Cohort N reflects participants whose cohort baseline date fell within the specified era. Initiation percentages are within-era. Lower initiation proportions in the 2017+ era partly reflect administrative censoring due to shorter calendar time available for follow-up rather than true reductions in treatment uptake. Median days to DMT for the 2017+ Other-race group is suppressed in accordance with the All of Us Data and Statistics Dissemination Policy. DMT, disease-modifying therapy.*

**Supplementary Table S7.** DMT initiation patterns by sex.

| **Sex** | **Cohort N (%)** | **Initiated, n (%)** | **Median days to DMT** |
| --- | --- | --- | --- |
| Female | 2,714 (77.4) | 1,221 (45.0) | 1,376 |
| Male | 781 (22.3) | 339 (43.4) | 997 |

*Participants with sex recorded as Unknown or Other (n=13) are not displayed in accordance with the All of Us Data and Statistics Dissemination Policy; these participants are included in cohort totals. The cohort baseline female-to-male ratio (approximately 3.5:1) is consistent with the established epidemiology of multiple sclerosis. Given the right-skewed distribution of time to first DMT, the median is the primary summary measure. DMT, disease-modifying therapy.*

**Supplementary Table S8.** Disease-modifying therapy initiation and first-line class in the 2017-onward subcohort, by race.

**Panel A.** Initiation, timing, and first-line class within the 2017+ subcohort.

| **Race group** | **N in 2017+** | **DMT initiators, n (%)** | **Median days to first DMT** | **Most common first-line class** |
| --- | --- | --- | --- | --- |
| White | 401 | 125 (31.2) | 176 | Infused high-efficacy |
| Black | 94 | 49 (52.1) | 200 | Infused high-efficacy |
| Other | 155 | 32 (20.6) | Suppressed | Infused high-efficacy |
| Overall | 650 | 206 (31.7) | 184 | Infused high-efficacy |

**Panel B.** Between-group differences in DMT initiation rate, all eras versus 2017+.

| **Comparison** | **All eras (pp)** | **2017+ (pp)** | **Direction** |
| --- | --- | --- | --- |
| Black vs. White | +3.9 | +21.0 | Widens |
| Other vs. White | −8.6 | −10.5 | Widens |
| Black vs. Other | +12.4 | +31.5 | Widens |

*pp = percentage points. All-era initiation rates are those reported in Table 1 (White 45.3%, Black 49.2%, Other 36.8%). The 2017+ subcohort comprises participants whose cohort baseline date fell on or after 1 January 2017; Panel A is consistent with the 2017+ row of Supplementary Table S6C. Median time to first DMT for the Other-race group is suppressed because the underlying cell contains fewer than 20 participants, per All of Us Controlled Tier reporting policy. Absolute within-era initiation rates in the 2017+ subcohort are depressed for every group by truncated observation time; the interpretable quantity in this table is the between-group difference within the same era, to which the shared truncation applies equally. Residual bias remains possible if follow-up duration within the 2017+ window differs systematically by race group. Median time to first DMT within the 2017+ subcohort is reported for completeness but should not be interpreted as evidence that the timing disparity narrowed, since in a truncated observation window only rapid initiators are observable and these medians are conditioned on short time-to-treatment by construction. Ethnicity was not separately stratified within the 2017+ subcohort: the expected number of Hispanic participants entering the cohort in 2017 or later is approximately 100, with roughly 26 initiators; an initiation rate for this subgroup would be disclosable, but median time to first DMT, class distribution, escalation, and discontinuation would fall below the n<20 reporting threshold. DMT, disease-modifying therapy.*
